# Labor market decisions after motherhood: The case of Barcelona ^1^^☆^

**DOI:** 10.1016/j.heliyon.2024.e25648

**Published:** 2024-02-13

**Authors:** Cristina de Gispert, Maite Vilalta, Paula Salinas

**Affiliations:** a Department of Economics, Av. Diagonal 690, Torre 6-Planta 2, Barcelona, 08034, Spain; b Universitat de Barcelona, Spain; c KSNET, Knowledge Sharing Network, Spain

**Keywords:** Gender inequality, Motherhood, Women's labour supply

## Abstract

This article analyses mothers' work decisions and their determinants during the first three years of their children's life, based on data from a survey of 1219 mothers in the Barcelona area during 2020. The factors affecting the probability of mothers reducing their working day or leaving their job after having a child are studied through a descriptive analysis, as well as by estimating a multinomial logic model. The results obtained indicate the relevance of the following aspects: the mother's income level, her level of education, the number of children and the fact of having the daily help of grandparents for childcare. The survey data show that the main reason mothers decide to reduce their working day or leave their job is to care for their children. These results are relevant for the design of childcare policies and work-life balance policies with the aim of avoiding gender inequalities in the future.

## 1 Introduction

Participation of women in the labour market has increased significantly in the last 30 years. Specifically, the OECD [1] average indicates that female employment rate between 1985 and 2015 has grown from 56% to 73% among women aged 25 to 54. Nonetheless, women's employment rate is still lower than that of men. Much of this difference between men and women is explained through the need for childcare within the family as shown in the study of Save The Children [2]. According to this report and based on data from EPA (Spanish Labour Force Survey) [3] the employment gender gap in Spain moves from 10 points between men and women without children to 22 points between men and women with children under 12. In terms of the unemployment rate, while for men it is higher among those without children (21.8% compared to 13.6%), the ratio is inverse for women (20.2% without children and 23.5% with children).

According to the report, women also face alignment difficulties indicated by their incidence of part-time contracts, especially among those with children. While only 6% of men with children work part-time, this percentage increases to 29% for women, with 47% of these latter doing so for childcare reasons compared to 7.7% of men. In the OECD, women's probability of part-time work is three times higher than men. Hupkau and Ruiz-Valenzuela (2021) [4] found that in the last decade, women with part-time contracts tripled compared to men (23% versus 7%).

The pandemic has negatively impacted female employment, especially because of the high number of women working in the services sector, which was severely affected by COVID-19. Women also bear a disproportionate burden of unpaid childcare and household chores, leading more women than men to leave the labour market, with adverse effects on economic growth. If female employment in Spain reached the levels of top ranked country Sweden, the economy would grow by 14%.^1^

This paper focuses on analysing women's work decisions in the first few years after giving birth. The study aims to understand the factors influencing women's preferences and choices regarding their labour market participation, as well as the decision that each type of family makes regarding labour market participation. The data used for the analysis come from a survey conducted in Barcelona and its metropolitan area, targeting mothers of children born between 2016 and 2019, with children aged 0–4 years at the time of the survey in 2020. The study explores how various family socioeconomic factors affects the likelihood of mothers reducing their working hours or leaving their jobs.

The decisions regarding labour participation in families are influenced by multiple factors. Previous research has highlighted that the availability of childcare services for children aged 0–3 years plays a crucial role in families' decisions to return to work, particularly for mothers (Nollenberger and Rodríguez-Planas, 2015; Carta and Rizzica, 2018; Baizán and Gonzalez, 2007) [[5], [6], [7]]. Other significant factors in determining labour market participation include the cost (Borra, 2010; Borra and Palma, 2009; Suarez, 2013) [[8], [9], [10]] and quality (Blau and Hagi, 1998) [11] of childcare services.

Besides the availability, cost, and quality of childcare services for children aged 0 to 3, the adaptability of these services to changing circumstances and varying needs, as well as the regional and familial disparities, are also crucial factors to consider (Blasco, 2018) [12]. The inflexibility of nursery schools, particularly regarding their schedules, has led to the emergence of different public and private initiatives aiming to address specific childcare demands. Some of these initiatives represent social innovations, involving novel organizational forms and family participation, such as family spaces, parenting groups, or day mothers. Despite being on a small scale, these socially innovative care services can significantly improve the quality of life and well-being for families.

It can be observed that decisions regarding labour participation in families and their demand for childcare services are interdependent and often made simultaneously, especially during the initial years after having a child. However, these decisions are complex and influenced by various factors, including parental income and education level, the child's age and health, and prevailing cultural values, among others. Moreover, gender dynamics in the division of domestic work play a significant role in determining who has more available time for paid work.^2^

Precisely, this article contributes to the analysis of this type of factors. Specifically, it aims to provide more evidence on what kind of decisions women make, in terms of their work, after becoming mothers and during the following years, focusing on the case of Barcelona and her metropolitan area. Conducting this type of study is essential to identify possible gender inequalities in the workplace. It is also important to understand in detail the causes of the wage gap between men and women, given the influence of maternity on their long-term employment trajectory. Finally, this study serves as a basis for developing more flexible labor policies and practices including the diversified provision of childcare services. Analyzing these decisions from different perspectives contributes to a deeper understanding of the challenges women face in the labor market after becoming mothers.

The document is structured as follows: In the second section, following this introduction, there is presented the literature review. In the third section we provide the details of the data collection and a description of the sample. The fourth section describes the methodology used to carry out the analysis. The fifth section focuses partially on describing the labour decisions of mothers during the first years of their children's lives, as well as showing which are the probabilities of making certain decisions regarding work by those mothers who worked full-time and with an indefinite contract before having a child. Finally, there are presented the main conclusions of the study and its policy implications.

## 2 Literature review

The conventional explanation for the demand for early childhood care services aligns with the theory of consumer behaviour. Families seek childcare services based on their preferences and constraints, such as budgetary and time limitations (Chaudry et al., 2010) [15]. Family preferences are influenced by factors like household composition and the parents' education level, with higher-educated parents valuing early education benefits more. The family's budgetary constraint is determined by household income and service prices, while the mother's employment situation mainly dictates the time constraint (García-Mainar et al., 2011) [16].

Furthermore, the decision regarding early childhood care involves not only whether to use childcare services but also which type of care to choose. This decision is influenced by preferences and the relative prices of different alternatives, creating a substitution relationship between formal and informal care services. According to the literature, families with higher education levels tend to prefer formal services due to their emphasis on child socialization and early stimulation (Del Boca and Vuri, 2007; Mamolo et al., 2011) [17,18]. The proximity of children centres or living close to relatives also impacts parents' decisions (Borra, 2010; Baizán and Gonzalez, 2007; Suárez, 2013; Borra and Palma, 2009) [[7], [8], [9], [10]]. Additionally, using a single form of childcare is found to be more efficient when there is more than one preschool-aged child at home (Del Boca and Vuri, 2007) [17].

### 2.1 Models of analysis of the demand for early childhood care services and labour participation

When families demand childcare services in the market, they do not only satisfy their usefulness as consumers of childcare services *per se* but also “buy” time that can be dedicated to the labour market. Therefore, this demand is closely related to the labour supply, especially that of mothers (Chaudry et al., 2010) [15]. In fact, many studies have considered the father's labor supply as a factor independent of the availability and use of childcare services, whose effect only conditions household income. However, Legazpe and Davia (2017) [19] for the Spanish case, dispense with this assumption of exogeneity arguing the change in family values and also considering the effects of the crisis on employment from 2008, affecting the availability of fathers (men) to take care of children and partially replacing the time of mothers' dedication.

The literature addressing the demand for childcare services in relation to labour market decisions has several lines of analysis. One first line focuses on the labour supply of mothers and considers care services in terms of the cost it entails for the family associated with the decision to work. That is, the demand for these services is completely determined by the decision of the family regarding the labour supply. This view simplifies a decision that is much more complicated because obviously the cost of care services is something endogenous that will depend on their quality as well as other attributes. Recognizing this endogenous relationship leads to the simultaneous determination of the demand for childcare services and female labour participation, which the second approach to the issue considers. This second line of analysis began with the work of Blau and Robins (1988) [20], Connely (1992) [21] and Ribar (1995) [22], and continued with the work of Del Boca and Vuri (2007) [17], Tekin (2007) [23], Van Gameren and Ooms (2009) [24] and Borra (2010) [8].

A third line incorporates the choice between the different available childcare types to the issues mentioned above. In this approach, the demand for these services may be explained by other additional factors, such as educational and developmental opportunities for the child. The work of Blau and Hagy (1998) [11] is the first one to analyse as a whole the mother's decision about: work, the decision to pay for care, the type of care chosen, and the demand for other attributes. The quality of these care services is considered from observable attributes such as: group size, teacher/student ratio, and service provider experience (although there are other unobservable ones such as energy and the motivation of the supplier).

In the case of Spain, the reach of education from 0 to 3 years of age is still limited compared to other countries. Thus, while in Spain the percentage of children under 3 years old enrolled in formal care institutions for at least 30 h a week is 20.6%, in Norway it is 46%, and in Denmark 69%, according to Eurostat data. However, in recent years, the offer of innovative alternative models has been added to the traditional institutionalized offer of education from 0 to 3 years old. Besides expanding the number of available places this latter has also expanded the variety of educational models available. Given that this phenomenon is very recent, and very geographically localized in certain areas, there is still no literature that analyses its impact on the labour market participation decisions of families or the schooling decisions of children from 0 to 3 years old.

### 2.2 Variables that determine the decisions for childcare and labour participation

According to the theoretical models presented, the main factors that would determine the decisions of families for demanding childcare services and about labour market participation are the cost of early childhood care services, their availability (*per se* and their distribution throughout the territory) and their affordability, as well as their quality. However, the empirical literature is not always conclusive regarding the relevance of these factors. It is possible to find studies that corroborate their relevance and others that contradict this theory.

Much of the empirical literature has focused on analysing the effects that the costs of care services have on their demand and the decisions by women in relation to the participation in the labour market. In general terms, the evidence shows that there is a negative relationship between the costs of childcare and the likelihood of participation in the labour market. For the case of Spain, Borra (2010) [6] estimates that the elasticity of labour participation to the price per hour of care services is between -0′81 and -0′94, a much higher elasticity than that estimated for other countries.

The availability of care services is another factor that can play a relevant role in women's work decisions. Nonetheless, few empirical works have focused on analysing the relationship between the geographical supply of care services and women's labour participation (Borra, 2010) [8]. Most studies include the availability of care services as a control variable (Han and Wladfogel, 2001 [25]; Del Boca and Vuri, 2007) [17]. The few studies that have focused on the analysis of accessibility to childcare services have found positive effects on women's labour force participation in the United States (Herbst and Barnow, 2008 [26]; Stolzenberg and Waite, 1984) [27], and nonsignificant effects in Germany (Kreyenfeld and Hank, 2000) [28]. A study from 2013 in the United States shows that the increase in nursery schools leads not only to an improvement in the academic results of children (around 1%), but also to an increase in the mother labour supply up to a 4.7% (Cascio and Schanzenbach, 2013) [29]. In Canada, an assessment of such policies points to an increase of up to 8 points in women's labour market participation (Haeck et al., 2015) [30]. The impact on the labour supply of mothers seems to be greater in the case of single mothers, one of the groups that find it most difficult to reconcile work and family life (Gelbach, 2002) [31]. In the case of Spain, Baizán and González (2007) [7] analyse the effects of the availability of care services on the labour participation among women. However, it does not have into account the endogeneity of the demand for childcare services, and it does not control for the levels of wages or the costs of the services.

In relation to the quality of services, the results are not conclusive either. Thus, while some studies conclude that the effects of service quality are not meaningful to explain the employment of women (Kimmel, 1998) [32], others obtain positive effects (Ribar, 1992, 1995) [[33], [22]].

When interpreting these results, it should be considered that these may vary significantly between different countries or regions due to labour market and welfare state characteristics, among other reasons (Cebrián et al., 2019) [34]. Thus, various studies have shown how the effects of changes in the prices of childcare services, or their availability will also depend on job market opportunities in each context (Berniell et al., 2023 [35]; Borrell-Porta et al., 2023 [36]). Cultural aspects, such as the willingness to delegate childcare to third parties, are also relevant. Taking care of children entails characteristics such as leisure; it is enjoyable and can have a diverting and social component, which in certain cultural settings increases the reputation of mothers and fathers as such. But it entails lots of unpaid time for dedication and therefore a significant opportunity cost. It is a mixture of the leisure and housework with no easy replacement within the market (Ribar, 1995 [22]; Pailhe and Solaz, 2008 [37]). Alesina and Giuliano (2010) [38] show that in countries such as Spain or Italy, where family ties are stronger, the participation rate of women is lower, and the time spent on domestic work and childcare is comparatively higher.

## 3 The data set

The data used are part of a more extensive research project (see footnote 1) aimed at analysing the labour trajectories of mothers with children under three years of age and the use of formal and informal early childhood care services. It focuses on the supply and demand of innovative childcare services. Consequently, it does combine quantitative and qualitative approaches, the latter focusing on interviews with mothers, as well as with policy makers and other stakeholders. These qualitative aspects are analyzed in Gallego & Maestripieri (2022) [39]. However, the present article provides merely quantitative results within the extended project.

The data were collected between July and September 2020, having conducted a pilot in March of the same year. The survey was distributed in a first phase using CAWI (Computer Aided Web Interviewing) methodology and, in a second phase, the CATI (Computer-Assisted Telephones Interviewing) methodology was chosen to compensate for the overrepresentation of mothers with university studies in the first phase. Finally, face-to-face interviews (CAPI) with families in a situation of extreme vulnerability were incorporated.

The final sample includes 1219 mothers who had at least one child between 1 and 4 years old at the moment of interview -born between 2016 and 2019-, while they could also have older children. Specifically, 66% of the cases belong to municipalities in the metropolitan area of Barcelona (808 observations with 524 from the city of Barcelona itself) and 34% correspond to municipalities in Catalonia that do not belong to the metropolitan area of Barcelona.

The survey is structured in blocks. Characteristics of the mother who responds; characteristics of the family unit; characteristics of the child for whom the questionnaire is answered; retrospective work history of the mother during the child's 0–3 years (category, working day, hours, salary); relationship between occupation and family organization; work history of the father; type of care/education of the child during the 0–3 years; assessment and knowledge of the educational function of the 0–3 years; 0–3 offer of the neighbourhood and informal education at home.

Table 1 in the appendix shows the descriptive statistics of each variable used in the econometric model.13.5% of the mothers in the sample are under 30 years old, 63.6% between 31 and 40 years old, and 22.9% over 40 years old. 77.9% of the mothers were born in Catalonia (42.3% were born in the same municipality where they currently reside and 35.5% in another municipality in Catalonia). 13.4% were born outside of Spain and 8.8% in another autonomous community (region) in Spain. Those born abroad come mostly from the American continent (58.3%) or the rest of Europe (31.3%). 8.6% were born in Africa and 1.8% in Asia.

68.3% of the interviewed mothers have a university degree or higher professional education (25.3% have a bachelor's degree; 22.9% have a master's degree and/or a doctorate; 20.1% have a higher professional education). 20.5% of the interviewed mothers have a baccalaureate degree or intermediate vocational training (10.3% baccalaureate; 10.3% intermediate vocational training). Those who have left school at the end of compulsory education represent the 10.5% (2.8% have completed primary school until the age of 14; 7.7% have completed secondary school until the age of 16). Only 0.7% of mothers have not finished compulsory education. These levels of education have been grouped into three categories to simplify the analysis: high, which includes mothers with a university degree (48,1%); medium, which includes mothers with high school, non-university higher education or vocational training (40,6%); and low, which includes mothers who have no studies or only have completed compulsory basic education (11,2%).

Almost all mothers, that is 92.4%, live with the father of their child; 3.5% are separated or divorced and do not live with a partner; 3.4% have never lived with the father of her child; 0.5% are separated or divorced from their child's father and live with a new partner; and 0.2% are widows.

Before the birth of the child the 92% of mothers worked. Out of these, 59.1% had an indefinite contract, 17.3% had a temporary contract, 12.2% were civil servants, 9.5% were self-employed and the remaining 1.9% did not have a formal contract. In addition, 81.7% of the women worked full-time (30 or more hours a week), while the rest worked part-time (mostly between 16 and 29 h a week).

Among the women who were working before having a child, 59% had a net annual income of less than 20,000 euros (1666 euros/month on average). Of these, 18.9% earned less than 9000 euros per year, which is less than 750 euros per month. Only 3.8% had a net annual income of more than 35,000 euros (approximately 3000 euros per month). Thus, 56.4% of mothers say that their income before the birth of their child contributed less than half of the total household income. In 3.9% of cases, the income of the mother was the only household income.

In terms of mobility, 80.7% of mothers have not changed neighbourhoods after having their child. The main reason for those having changed (19.3%) has been, firstly, the price of housing (40.4%) and secondly, organizational reasons regarding family (28.5%). There is only a 5.5% that did it for labour purposes and 4.7% because of the school supply in the neighbourhood.

## 4 Methodology

The survey data used in this study allows us to analyse the labour decisions of the mothers, their decisions regarding the type of care for their children, and the relationship between these two decisions during the first 3 years of the children's lives. A multinomial logit model is estimated (with the statistical package STATA) in order to analyse the probability for mothers to decide to reduce their workday or leave their jobs after having a child, as well as the determinants of these decisions. Thus, the analysis focuses mainly on studying how the probabilities of reducing the workday or leaving work are influenced by the socioeconomic characteristics of mothers, especially their education and income levels before having the child.

In the specified model, the dependent variable (Y) is, therefore, the labour decision of the mothers, which includes 3 categories or alternatives (j): firstly, keep the workday the same as the one before having the child. Secondly, reduce their workday; and the third, quit their job. The decision of mothers to reduce their workday or quit their jobs may be influenced by various socioeconomic factors and their family characteristics (X). More precisely, we include in the model the characteristics of the mother, including her age, her level of education or whether she is an immigrant coming from outside of Europe. We also include the characteristics of the mother's work before the birth of the child. Such as the type of workday (partial-time with less than 15 h, partial-time between 16 and 30 h, or full-time with over 30 h per week); the employment situation (civil servant or equivalent, indefinite contract, temporary contract, self-employed, or without a formal contract); and the income level. Furthermore, we include the characteristics of the couple such as their level of education, whether they are immigrants from outside Europe, and their income level. Finally, other family characteristics such as the number of children under the age of 18, whether they live with the child's father, whether they live with grandparents, whether the domestic work is divided equally between the mother and her partner, and whether grandparents care for the child on a daily basis ara also included. We also include two variables that allow us to approximate the economic situation of the family: if they have a home with a mortgage and if before the birth of the child, they could face an unforeseen circumstance worth 700 euros.

Our interest is focused on analysing how the different X variables alter the probabilities of mothers making some work decisions over others after the birth of their sons and daughters. In the multinomial logit model, the probabilities can be expressed as the following equation (1):

(1) P (Y = j|X_1_,X_2_, …, X_k_) = P(Y = j|X)

where j = 1,2,3 represent the three alternative labour decisions of mothers. To carry out an estimation, the base category is the option of keeping the same workday as before the birth of the child. The estimated model is the following:

$$\ln\left(\frac{P\left(Y={reduce\;workday}_{e}\right)}{P\left(Y={keep\;workday}_{e}\right)}\right)=\beta_{10}^{e}+\beta_{1k}^{e}X_{ik}^{e}$$

$$\ln\left(\frac{P\left(Y={quit\;job}_{e}\right)}{P\left(Y={keep\;workday}_{e}\right)}\right)=\beta_{20}^{e}+\beta_{21}^{e}X_{i}^{e}$$

where $\beta_{ak}^{e}$ are the regression coefficients for each of the two estimated equations a, and for each child age (e). These coefficients represent the logarithm of the ratio of probabilities in case of a unitary variation of the variable $X_{ik}^{e}$. That is, how the logarithm of the relative probability of reducing the workday or quitting the job with respect to keeping the workday, when there is a change in the variable $X_{ik}^{e}$. Based on these parameters, the probability ratio of reducing the workday or leaving the job in relation to maintain the workday can be calculated, which is also called relative risk. Likewise, the estimated probabilities can also be calculated. They make it easier to interpret the results of the model.

Finally, the estimated probabilities of reducing the workday or leaving the job for different mother profiles are also shown. Specifically, these probabilities are calculated for mothers who before having a child worked full-time and with an indefinite contract, depending on their level of education and their income level. It is also calculated how these probabilities vary depending on the number of children and whether grandparents take care of the children on a daily basis or not. When calculating these probabilities, the remaining variables are kept at their value for each observation. Then, the estimated probabilities are averaged for all observations.

## 5 Labour decisions of mothers during the first years of life of their children

This section presents the main results obtained from the survey on the employment situation of mothers, from the time they have the child up to the three first years of life. This is a crucial stage for both the development of the child and for the future trajectory of women in the labour market. The analysis is approached, firstly, at a descriptive level to understand which decisions have been taken and why; and secondly, with a regression analysis to know with what probability certain decisions will be taken, such as working or not working, or the option of reducing the working day, depending on certain socioeconomic characteristics of the family.

### 5.1 Description of the labour decisions of mothers and their motivations

**Graph 1:**
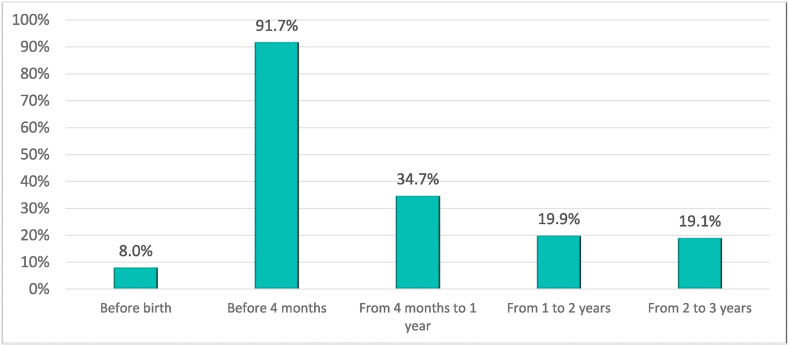
Percentage of mothers who do not work before and after having a child.

*Source:* own elaboration from survey data

As official statistics show, the employment rate of women is still lower than that of men. In the sample, out of 1219 families, 8% of women did not work before the birth of their child compared to 4.1% in the case of their partners. Graph 1 shows how this percentage increases after having children, up to 34.7% between 4 months and one year later, and it remains above 19% between one and three years later. That is, 28.7% of mothers stop working during the first year of the child's life, and 14% remain unemployed when the child is 3 years old. According to the survey results, 70% of mothers who do not work after having children do so for reasons related to childcare.

**Graph 2:**
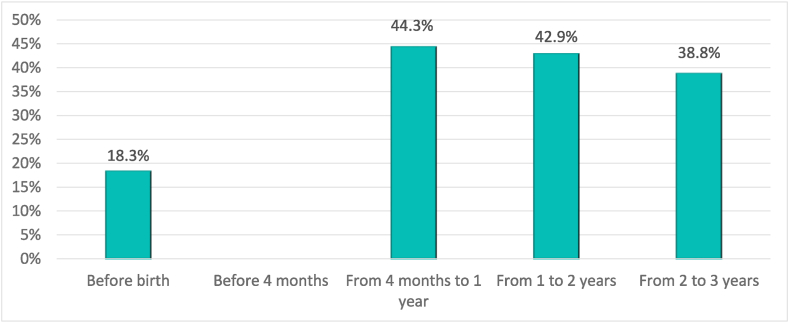
Percentage of mothers who work part time before and after having a child.

*Source:* own elaboration from survey data

It should also be noted that a high percentage of women does not quit their jobs but do reduce their working hours after having children. Specifically, Graph 2 shows that the percentage of women who work part-time increases from 18.3% before having children to 44.3% and 42.9% during the first and second year after having children, respectively. Between the second and third year, this percentage drops to 38.8%, which is still significantly higher than before having children.

According to the survey answers, more than 85% of women work voluntarily part-time, compared to 55% who worked voluntarily before having children. In fact, almost 50% of working mothers say that they would have preferred to work less in order to spend more time with their child, and 32% of them state that despite having a quality resource to care for their child, they would not have worked more hours. In comparison, 6.1% say that if they had found a reliable and affordable quality resource, they would have worked more hours.

**Graph 3:**
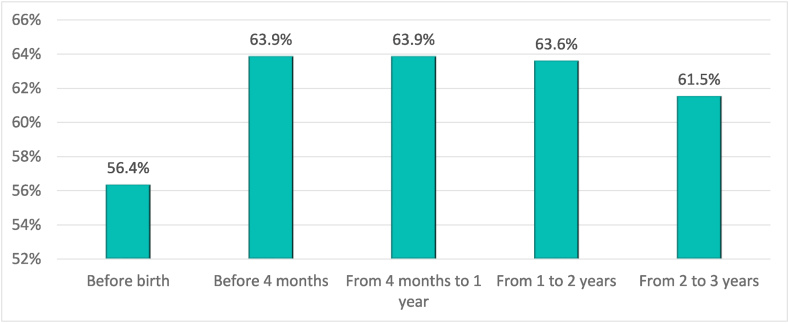
Percentage of mothers whose income accounts for less than 50% of household income before and after having a child.

*Source:* own elaboration from survey data

This reduction in the labour intensity of mothers implies an increase in the number of mothers with no or reduced income, as well as a reduction in the weight they represent in the total income of the family economy (Graph 3). In fact, several studies^3^ point out that the origin of the wage gap lies precisely in the reduction of work dedication among women after becoming mothers. This includes either reducing the number of working days or moving to part-time jobs or temporary contracts. Therefore, economic inequality between women and men increases after having children.

### 5.2 Family characteristics that determine mother's working decisions

Table 2 in the appendix presents the results obtained from the estimation of the multinomial logit model described in section 4, to analyse the extent to which the socioeconomic characteristics of mothers and their families can condition their work decisions.

As it can be seen in Table 2, the probability of quitting work or reducing the working day is greater the lower the income level. For example, the probability of stopping work during the first year of the child's life is, on average, 19.9 percentage points higher for mothers with no income than for mothers with an income of more than 25,000 euros per year; and 12.8% percentage points higher for mothers with an income of less than 9000 euros.

Also, mothers with no education have a 12.2 percentage point higher probability of leaving work than mothers with a university education in the first year of the child's life; 19.9 percentage points higher between one and two years of age; and 15.1 percentage points higher between two and three years of age. In this case, no differences are observed in the probability of reducing the working day.

Self-employed working mothers are more likely to reduce their working day during the first year after birth (15.7% more), and less likely to leave their job (31% less); and these probabilities decrease to zero after one year. This can be interpreted as meaning that the flexibility that self-employment can offer favours mothers not leaving the labour market, although they are less likely to reduce their working day during the first year.

Being able to count on the grandparents on a daily basis to take care of the children reduces the probability of leaving work by 32.8 percentage points in the first year, 19.8 percentage points in the second year, and 9.4 percentage points between 2 and 3 years. It is interesting to see that when this variable is included, the immigrant variable ceases to be significant. That is, when the family network is not considered, immigrant mothers are more likely to leave work. But when the variable that the grandparents take care of the children every day is considered, the immigrant variable is no longer significant.

### 5.3 Probability of leaving a job or reducing working hours as a function of income and studies level

Focusing on mothers who worked full-time and with an indefinite contract before having a child, we estimate the probabilities of leaving their jobs or reducing their working hours, according to different levels of income and studies, and maintaining constant the rest of variables, (these probabilities are detailed in Table 3 of the appendix). Graph 4a, Graph 4b shows that the probability of leaving work or reducing working hours is greater the lower the income level. Specifically, it is observed that the probability of reducing the working day decreases especially above the threshold of 19,000 euros, up to 19% (child between 1 and 2 years) and 11% (child between 2 and 3 years), when the mother's level of education is high. The probability that mothers stop working also decreases as the level of income increases, despite the fact that it achieves the minimum value for an income between 14,000 and 19,000 euros, and then the probability picks up again.

**Graph 4a:**
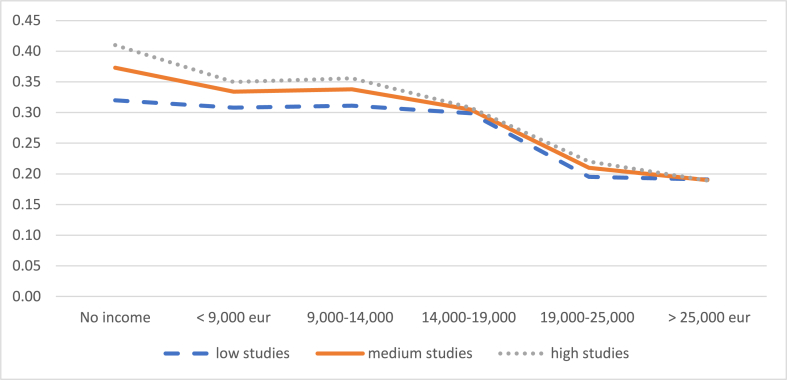
Probability of reducing the working day when the child is between 1 and 2 years old. Evolution by income level. (%). **Panel A**.

**Graph 4b:**
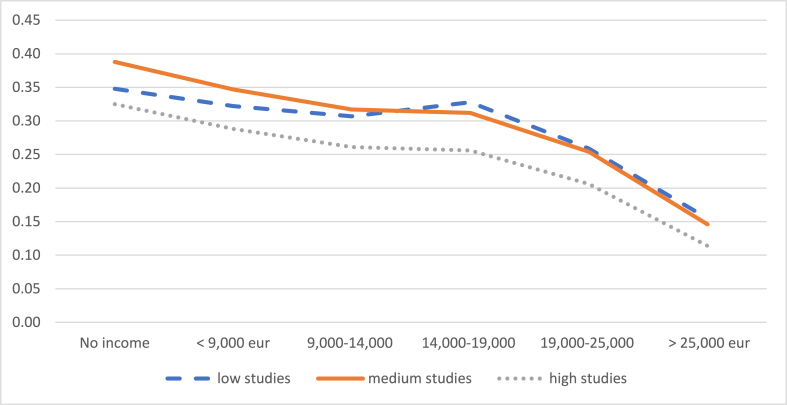
Probability of reducing the working day when the child is between 2 and 3 years old. Evolution by income level. (%). **Panel B**.

*Source:* own elaboration from survey data

If we compare the probability that a mother who earns the minimum wage (13,300/year by 2020) will reduce her working day according to her level of education - either when the child is between 1 and 2 years old (Graph 4. Panel A) or when the child is between 2 and 3 (Graph 4. Panel B) - the difference ranges from 31.1% (low education) to 35.6% (high education) in the first case, to 26.1% (high education) to 31.7% (medium education) in the second case. Therefore, comparatively, the level of income is a factor that differentiates the probability of reducing the working day more than the level of education, probably because it determines the opportunity cost of working fewer hours and because a higher level of education does not always imply a higher income. When the child is between 2 and 3 years old, for an average level of education of the mother, the probability of reducing the working day differs by 20 percentage points between those who earn less than 9000 euros (35%) and those who earn more than 25,000 euros (15%) (Graph 4a, Graph 4b. Panel B).

As for the probability of leaving the job (Graph 5), it is observed that, for an average level of education of the mother, this probability ranges from 25% (no income) to 9% (more than 25,000 euros) when the child is 1–2 years old (Graph 5a, Graph 5b. Panel A). From 16% (no income) to 9% (more than 25,000 euros) when the child is 2–3 years old (Graph 5. Panel B). In this case, having less education is clearly a factor that increases the probability of making this decision from 34% (no income) to 23% (more than 25,000 euros).

**Graph 5a:**
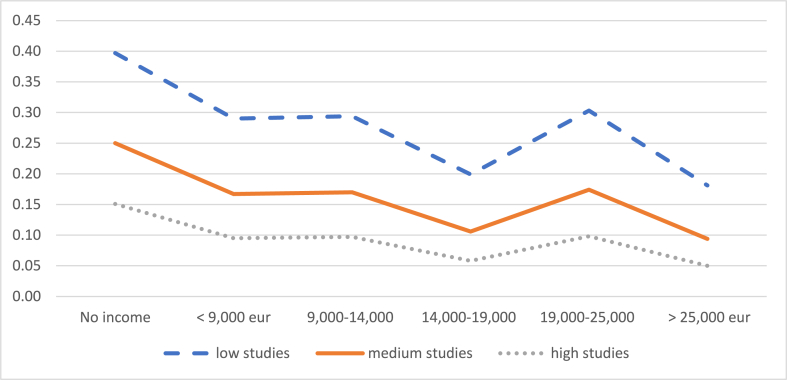
Probability of leaving work when the child is between 1 and 2 years old. Evolution by income level. (%). Panel **A**.

**Graph 5b:**
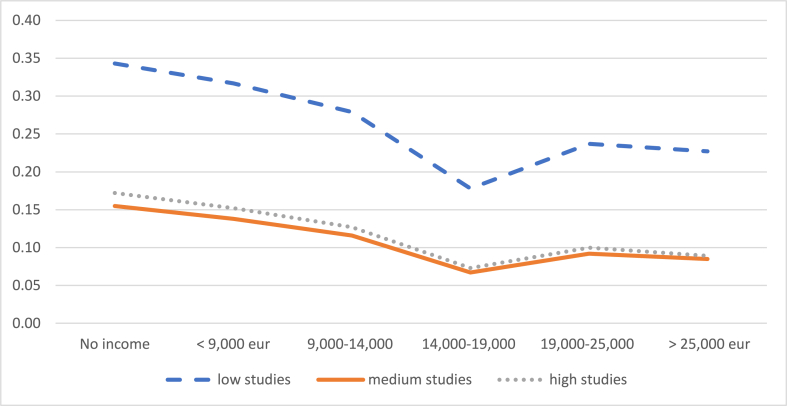
Probability of leaving work when the child is between 2 and 3 years old. Evolution by income level. (%). Panel **B**.

Source: own elaboration from survey data

Therefore, inequality does not increase equally for all mothers. According to these results, it is women with lower income levels and lower levels of education who are more likely to leave their jobs or reduce their working hours and, therefore, those whose income levels will be more affected after motherhood.

### 5.4 Probability of leaving a job or reducing working hours as a function of other family characteristics

**Graph 6:**
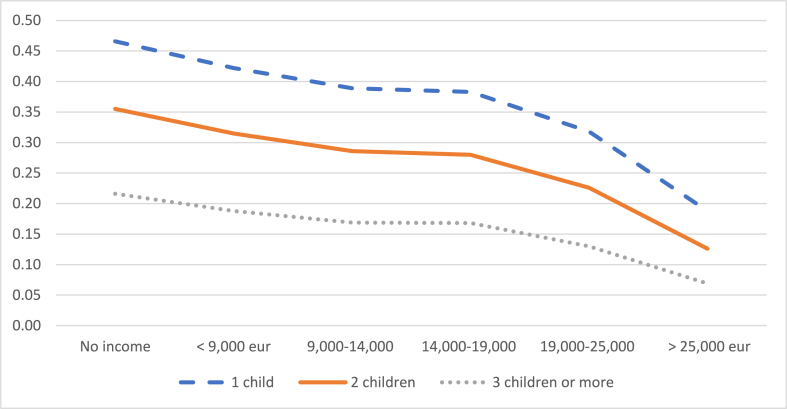
Probability of reducing the working day when the child is between 2 and 3 years old according to number of children. (%).

Source: own elaboration from survey data

**Graph 7:**
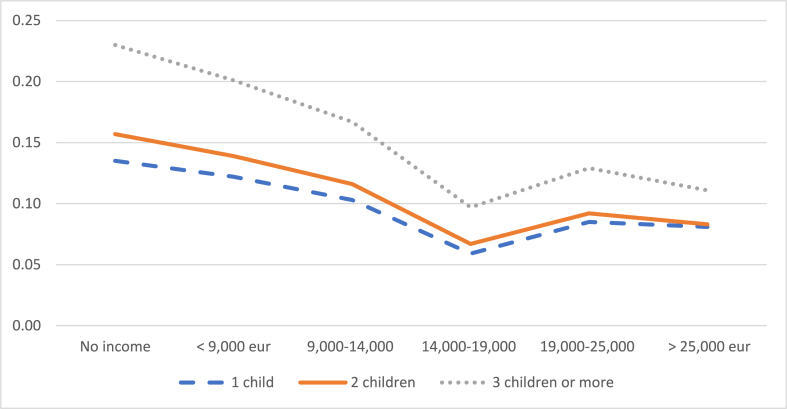
Probability of leaving work when the child is between 2 and 3 years old according to number of children. (%). Note: these probabilities are calculated for an average level of studies of the mother.

Source: own elaboration from survey data

Note: these probabilities are calculated for an average level of studies of the mother.

The probability of reducing working hours decreases with the number of children, although the probability of leaving work increases. Specifically, Graph 6 shows how a mother with an average level of education who earns the IMW has a 39% probability of reducing her working day if she has only one child, while this probability drops to 17% if she has three or more children. When the mother has an income of more than 25,000 euros/year and three or more children, the probability that she will decide to reduce her working day is only 7%. On the other hand, Graph 7 shows that the probability of leaving work if she has only one child is 12% when her income is less than 9000 euros and increases to 20% when she has three or more children. When the mother's income exceeds 25,000 euros/year, the probability of leaving the job rises from 8% for mothers with one child to 11% for mothers with three or more children.

**Graph 8:**
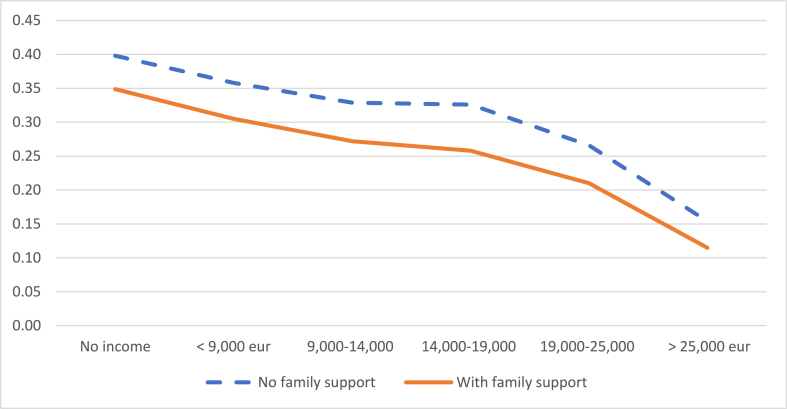
Probability of reducing the working day when the child is between 2 and 3 years old according to the daily help from grandparents. (%). Note: these probabilities are calculated for an average level of studies of the mother.

Source: own elaboration from survey data

**Graph 9:**
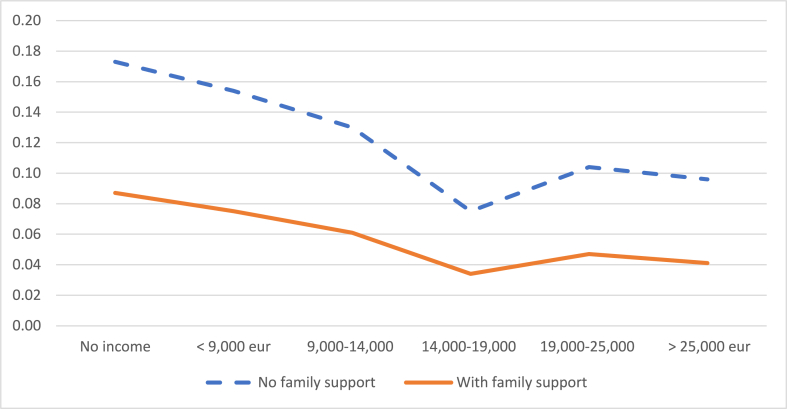
Probability of leaving work when the child is between 2 and 3 years old according to the daily help from grandparents. (%). Note: these probabilities are calculated for an average level of studies of the mother.

Source: own elaboration from survey data

Finally, having the daily support of the grandparents to take care of the children is also a factor that influences the decision to reduce the working day and the decision to leave work. Not having this help increases the probability of the mother reducing her working day by 5%, on average, compared to the situation in which the grandparents do collaborate (Graph 8). Likewise, not having this help increases the probability that the mother leaves work by 6.5%, on average, compared to the situation in which the grandparents do collaborate (Graph 9).

It is interesting to see that when “family support” variable is included in the model, the immigrant variable ceases to be significant. That is, when the family network is not considered, immigrant mothers are more likely to leave work. But when the variable that the grandparents take care of the children every day is considered, the immigrant variable is no longer significant (see Table 2 in the appendix).

## 6 Conclusions and policy implications

This article analyses mothers' work decisions and their determinants during the first three years of their children's life, based on data from a survey of 1219 mothers in the Barcelona area during 2020. The factors affecting the probability of mothers reducing their working day or leaving their job after having a child are studied through a descriptive analysis, as well as by estimating a multinomial logic model. Modelling these decisions is challenging due to the complexity arising from diverse family and work situations, as well as heterogeneous preferences that lack appropriate institutional responses.

A correct interpretation of the results obtained requires taking into account some limitations. It is an analysis at a moment in time (just before the pandemic and, therefore, in a different context). We still need to work on how it affects the total supply of health care services, both in number and in typology. The sample includes observations from the metropolitan area of Barcelona and from outside the area (in the latter case less so); it would be interesting to analyse these samples separately to detect differential aspects related, for example, to the territory urban or rural. But this would require a larger and more diversified sample size.

In the analysis, we have seen that over 14% of mothers working before the birth of their child stop working and around 30% reduce their working hours. The main reason for these changes is taking care of their sons and daughters. Additionally, 50% of working mothers claim to have preferred to work less so they could spend more time with their children. These figures only corroborate what the literature tells us: decisions about the model of care for children aged 0 to 3 are related and determined by the work decisions of mothers and vice versa.

The analysis reveals that over 14% of mothers working before childbirth stop working, and around 30% reduce their working hours, mainly due to childcare responsibilities. 50% of the working mothers prefer to work less to spend more time with their children, further corroborating previous literature outcomes: decisions about childcare models for children aged 0 to 3 are related and determined by work decisions of mothers and vice versa.

The decrease in work intensity among mothers leads to income loss, affecting the family economy and contributing to greater dependence and job instability. Studies suggest this may exacerbate the wage gap between men and women over time. In addition, lower-income and less-educated mothers are more likely to leave work or alter their employment status, which demonstrates that inequality does not increase equally in all families. According to these results, it is women with lower income levels and lower levels of education who are more likely to leave their jobs or reduce their working hours and, therefore, those whose income levels will be more affected after motherhood. Additionally, the results obtained are consistent with the theory of human capital, since their level of education, work experience, and skills influences the labor decisions of women after maternity.

We are then facing a problem that is characterized mainly by its heterogeneity and inequality. This implies a political need to face diverse and heterogeneous situations with varied and appropriate responses. In other words, uniform policies cannot be applied, nor can be applied equal solutions to unequal needs as they will not work. Moreover, it should be borne in mind that when governments try to respond to the diverse preferences of citizens, generally this translates into a common increase in well-being.

Therefore, there is a need for coordinated, transversal, designed, and implemented public policies from different levels of government to address this issue. The aim should be to ensure that we do not reach such an unequal starting point (childbirth) but to ensure that the first years of children's lives do not reinforce these inequalities. In other words, that motherhood does not increase the existing inequalities.

In brief, we must deepen our understanding of family decision-making process, especially among women, and address existing heterogeneities to design effective policies that reduce inequalities. It is essential to emphasize so-called socially induced innovative care services, which prioritize children's interests and complement traditional offerings. However, currently, these initiatives have little presence, probably due to the great administrative and economic difficulties faced by this type of project in Spain.^4^

Most of these innovative care services are not officially recognized as childcare services, creating mistrust among families. In addition, families using these services cannot access certain advantages, like tax relief in the Spanish Personal Income Tax for care services for children aged 0–3 years. Finally, these services operate on a small scale with low ratios, hindering their economic sustainability as private centres. The unit costs of such services should be explored and compared with nursery schools. Thus, evaluating their impact on children and families' welfare, as well as women's work participation is also crucial.

In conclusion, funding and governance of early childhood care must be addressed to ensure clear alignment with the competencies and responsibilities of each level of government. Obviously, the improvements require legal changes that are often slower than desirable. However, it should be noted that very interesting initiatives are being carried out in this direction, such as, for example, the implementation of the EU directive on Work-Life balance (2019)^5^ and the pension reform being implemented in Spain.^6^

## Ethic declarations

This study is part of a more global research project funded by the RecerCaixa program, promoted by the private Foundation "la Caixa" and the Catalan Association of Public Universities (ACUP). Grant number (2017ACUP04).

## Ethical approval

This article does not contain any studies with animals performed by any of the authors. All procedures performed in data collection and survey involving human participants were in accordance with the standards of ethical norms.

## Data availability

The data are available upon request of the author and provided that the request is duly justified for research purposes.

## CRediT authorship contribution statement

**Cristina de Gispert:** Writing – review & editing, Writing – original draft, Conceptualization, Data curation, Formal analysis, Investigation, Project administration, Resources, Software, Supervision, Validation, Visualization. **Maite Vilalta:** Writing – review & editing, Writing – original draft, Conceptualization, Data curation, Formal analysis, Funding acquisition, Investigation, Resources, Software, Validation, Visualization. **Paula Salinas:** Writing – review & editing, Writing – original draft, Conceptualization, Data curation, Formal analysis, Investigation, Methodology.

## Declaration of competing interest

The authors declare the following financial interests/personal relationships which may be considered as potential competing interests: The authors declare they have no conflict of interest.

Cristina de Gispert, Maite Vilalta and Paula Salinas reports financial support was provided by Fundación La Caixa.

## Figures and Tables

**Table 1:** Descriptive analysis of model variables

**Variable**	**Observations**	**Average**	**Standard Deviation**	**Min**	**Max**
Labor decision	1121	0.843	0.869	0	2
Mother's age	1219	36,648	5915	19	60
Mother born outside Europe	1219	0.092	0.288	0	1
Mother's level of education					
Low	1219	0.112	0.315	0	1
Medium	1219	0.406	0.491	0	1
High	1219	0.481	0.499	0	1
Type of mother's workday (before birth of the baby)					
Complete (more than 30 h/week)	1121	0.817	0.387	0	1
Partial (between 16 and 30 h/week)	1121	0.152	0.359	0	1
Partial (less than 15 h/week)	1121	0.030	0.171	0	1
Work situation of the mother (before birth of the baby)					
Civil servant or equivalent	1121	0.122	0.327	0	1
Permanent contract	1121	0.590	0.491	0	1
Temporary contract	1121	0.173	0.378	0	1
Self-employment	1121	0.095	0.293	0	1
No formal contract	1121	0.019	0.136	0	1
Mother's income level (before birth of the baby)					
No income	1148	0.113	0.317	0	1
Up to 9000 euros/year	1148	0.189	0.391	0	1
From 9001 to 14,000 euros/year	1148	0.182	0.386	0	1
From 14,001 to 19,000 euros/year	1148	0.218	0.412	0	1
From 19,001 to 24,000 euros/year	1148	0.192	0.393	0	1
More than 24,000 euros/year	1148	0.105	0.307	0	1
Partner's level of education					
Low	1162	0.170	0.376	0	1
Medium	1162	0.404	0.491	0	1
High	1162	0.425	0.494	0	1
Partner from outside Europe	1162	0.095	0.292	0	1
Income level of the partner					
Up to 14,000 euros/year	1141	0.166	0.372	0	1
From 14,001 to 19,000 euros/year	1141	0.173	0.378	0	1
From 19,001 to 25,000 euros/year	1141	0.233	0.423	0	1
From 25,001 to 30,000 euros/year	1141	0.224	0.417	0	1
More than 30,000 euros/year	1141	0.202	0.402	0	1
Children under 18					
1 child	1219	0.503	0.500	0	1
2 children	1219	0.417	0.493	0	1
3 or more children	1219	0.080	0.272	0	1
Other family characteristics					
She lives with the child's father	1219	0.924	0.265	0	1
She lives with the child's grandparents	1219	0.045	0.207	0	1
Grandparents take care of the child on a daily basis	1219	0.219	0.413	0	1
Equal distribution of housework	1219	4143	2556	0	10
Home with mortgage	1219	0.442	0.496	0	1
No ability to meet unforeseen expenses of 700 euros	1219	0.283	0.450	0	1

**Table 2:** Estimated probability of choosing each labor option for each level of explanatory variable, keeping all other variables constant

Child age:		Between 4 months and 1 year			Between 1 and 2 years			Between 2 and 3 years	
	(1)	(2)	(3)	(4)	(5)	(6)	(7)	(8)	(9)
VARIABLES	Keep working	Reduce working hours	Stop working	Keep working	Reduce working hours	Stop working	Keep working	Reduce working hours	Stop working
Mother's age	0.002	−0.001	−0.001	−0.001	−0.002	0.002	0.004	−0.003	−0.001
	(0.003)	(0.002)	(0.003)	(0.003)	(0.003)	(0.002)	(0.003)	(0.003)	(0.002)
Mother's level of education (Base: High):									
Low	−0.083	−0.039	0.122 **	−0.170 **	−0.029	0.199 ***	−0.196 ***	0.045	0.151 **
	(0.062)	(0.057)	(0.059)	(0.067)	(0.065)	(0.058)	(0.076)	(0.070)	(0.068)
Medium	−0.046	−0.013	0.059	−0.064	−0.012	0.076 ***	−0.038	0.046	−0.008
	(0.038)	(0.032)	(0.037)	(0.041)	(0.037)	(0.029)	(0.046)	(0.040)	(0.031)
Mother's workday before birth (Base: >30 h per week)									
Between 16 and 29 h a week	0.174 ***	−0.192 ***	0.017	0.243 ***	−0.256 ***	0.013	0.207 ***	−0.243 ***	0.036
	(0.039)	(0.025)	(0.037)	(0.036)	(0.025)	(0.031)	(0.042)	(0.025)	(0.037)
Less than 15 h a week	0.269 ***	−0.261 ***	−0.008	0.231 ***	−0.311 ***	0.080	0.146	−0.288 ***	0.142
	(0.092)	(0.015)	(0.092)	(0.076)	(0.017)	(0.074)	(0.098)	(0.018)	(0.098)
Mother's employment status before birth (Base: no formal contract):									
Civil servant or equivalent	−0.368 **	0.098	0.270 ***	−0.183	0.127	0.056	−0.129	0.235 *	−0.106
	(0.143)	(0.118)	(0.104)	(0.151)	(0.142)	(0.069)	(0.166)	(0.128)	(0.118)
Indefinite employment contract	−0.132	0.090	0.043	−0.032	0.009	0.023	−0.024	0.096	−0.071
	(0.138)	(0.113)	(0.095)	(0.145)	(0.135)	(0.060)	(0.158)	(0.117)	(0.113)
Temporary employment contract	−0.228 *	0.050	0.179 *	−0.071	−0.050	0.121 *	−0.033	0.051	−0.019
	(0.139)	(0.115)	(0.097)	(0.146)	(0.136)	(0.063)	(0.160)	(0.120)	(0.116)
Self-employed	−0.215	0.255 **	−0.040	−0.121	0.125	−0.005	−0.093	0.198	−0.105
	(0.145)	(0.123)	(0.099)	(0.152)	(0.144)	(0.065)	(0.166)	(0.130)	(0.116)
Mother's income before birth (Base: more than 25,000 eur)									
No income	−0.325 ***	0.126 *	0.199 ***	−0.325 ***	0.170 **	0.155 **	−0.294 ***	0.198 **	0.096
	(0.079)	(0.072)	(0.075)	(0.086)	(0.083)	(0.062)	(0.099)	(0.091)	(0.072)
Up to 9000 euros	−0.202 ***	0.074	0.128 **	−0.205 ***	0.131 **	0.074	−0.237 ***	0.165 ***	0.073
	(0.062)	(0.046)	(0.060)	(0.064)	(0.054)	(0.047)	(0.071)	(0.057)	(0.056)
From 9001 to 14,000 euros	−0.207 ***	0.107 **	0.099 *	−0.212 ***	0.135 ***	0.077 *	−0.185 ***	0.141 ***	0.044
	(0.059)	(0.043)	(0.055)	(0.061)	(0.049)	(0.047)	(0.068)	(0.051)	(0.053)
From 14,001 to 19,000 euros	−0.157 ***	0.107 ***	0.050	−0.117 **	0.102 **	0.015	−0.120 **	0.137 ***	−0.017
	(0.056)	(0.039)	(0.050)	(0.056)	(0.045)	(0.040)	(0.061)	(0.047)	(0.044)
From 19,001 to 25,000 euros	−0.137 **	0.075 *	0.061	−0.096 *	0.021	0.075 *	−0.103 *	0.089 **	0.013
	(0.056)	(0.039)	(0.049)	(0.056)	(0.042)	(0.043)	(0.060)	(0.045)	(0.046)
Level of education of the partner (Base: High)									
Low	0.084	0.024	−0.107 **	0.105 **	−0.026	−0.080 **	0.136 **	−0.079	−0.057
	(0.053)	(0.046)	(0.045)	(0.052)	(0.048)	(0.034)	(0.056)	(0.051)	(0.038)
Medium	0.003	0.029	−0.032	0.018	0.003	−0.021	0.032	−0.041	0.009
	(0.037)	(0.032)	(0.036)	(0.040)	(0.035)	(0.032)	(0.045)	(0.041)	(0.033)
Income of the partner before birth (Base: more than 35,000 eur)									
Up to 14,000 euros	0.102 *	−0.111 **	0.010	0.049	−0.125 **	0.076	0.011	−0.055	0.044
	(0.061)	(0.047)	(0.059)	(0.064)	(0.054)	(0.048)	(0.074)	(0.064)	(0.053)
From 14,001 to 19,000 euros	0.081	−0.036	−0.045	−0.015	−0.023	0.038	−0.011	0.036	−0.025
	(0.057)	(0.049)	(0.054)	(0.061)	(0.056)	(0.043)	(0.070)	(0.064)	(0.044)
From 19,001 to 25,000 euros	0.068	−0.023	−0.045	0.009	−0.039	0.030	0.024	−0.025	0.001
	(0.050)	(0.043)	(0.047)	(0.054)	(0.048)	(0.038)	(0.062)	(0.053)	(0.042)
From 25,001 to 35,000 euros	−0.019	−0.003	0.021	−0.067	−0.020	0.087 **	−0.045	−0.035	0.079 *
	(0.048)	(0.042)	(0.045)	(0.052)	(0.047)	(0.037)	(0.058)	(0.050)	(0.042)
Other economic variables:									
Home ownership with mortgage	0.026	0.017	−0.043	0.014	−0.006	−0.008	−0.009	0.054 *	−0.045 *
	(0.030)	(0.026)	(0.028)	(0.032)	(0.028)	(0.024)	(0.036)	(0.031)	(0.025)
Ability to meet unforeseen expenses of 700 euros	−0.012	0.021	−0.009	−0.030	0.005	0.025	−0.027	0.002	0.025
	(0.038)	(0.035)	(0.032)	(0.039)	(0.037)	(0.026)	(0.043)	(0.038)	(0.031)
Migratory origin:									
Mother born outside Europe	−0.082	0.015	0.067	−0.049	0.074	−0.025	−0.067	0.040	0.027
	(0.062)	(0.057)	(0.055)	(0.066)	(0.063)	(0.034)	(0.072)	(0.064)	(0.049)
Mother's partner born outside Europe	0.016	−0.024	0.008	−0.035	−0.049	0.083 *	0.022	−0.012	−0.010
	(0.059)	(0.053)	(0.049)	(0.058)	(0.052)	(0.044)	(0.063)	(0.059)	(0.041)
Family structure:									
2 children between 0 and 18 years old	0.112 ***	−0.075 ***	−0.036	0.131 ***	−0.093 ***	−0.037	0.073 **	−0.081 **	0.008
	(0.031)	(0.026)	(0.028)	(0.032)	(0.029)	(0.023)	(0.037)	(0.032)	(0.027)
3 or more children between 0 and 18 years old	0.245 ***	−0.155 ***	−0.091 *	0.170 ***	−0.180 ***	0.010	0.114 *	−0.168 ***	0.054
	(0.055)	(0.042)	(0.049)	(0.059)	(0.048)	(0.048)	(0.062)	(0.047)	(0.050)
She lives with the child's father	−0.082	0.017	0.065	−0.077	−0.022	0.099 ***	0.010	−0.043	0.033
	(0.093)	(0.075)	(0.076)	(0.087)	(0.083)	(0.035)	(0.105)	(0.093)	(0.066)
She lives with the child's grandparents	−0.060	0.077	−0.017	0.096	0.004	−0.100 ***	0.036	0.029	−0.066
	(0.101)	(0.101)	(0.089)	(0.100)	(0.096)	(0.038)	(0.116)	(0.109)	(0.046)
Grandparents take care of the child on a daily basis	0.285 ***	0.043	−0.328 ***	0.183 ***	0.015	−0.198 ***	0.134 ***	−0.040	−0.094 ***
	(0.034)	(0.029)	(0.040)	(0.040)	(0.033)	(0.037)	(0.043)	(0.037)	(0.034)
Equal distribution of housework	0.027 ***	0.003	−0.029 ***	0.029 ***	−0.010 *	−0.019 ***	0.031 ***	−0.021 ***	−0.010 **
	(0.006)	(0.005)	(0.006)	(0.006)	(0.006)	(0.005)	(0.007)	(0.006)	(0.005)
Observations	1056	1056	1056	940	940	940	744	744	744

Standard errors in parentheses.

***p < 0.01, **p < 0.05, *p < 0.1.

**Table 3:** Probability of leaving work or reducing working hours for full-time working mothers with an indefinite contract 3a. Probability of reducing working hours or leaving work depending on the level of education and the level of income, between the year and the child's 2 years 3b. Probability of reducing working hours or leaving work depending on the level of education and the level of income, between 2 and 3 years of the child 3c. Probability of reducing working hours or leaving work depending on the number of children and the level of income, 2–3 years of age, average level of education. 3d. Probability of reducing working hours or leaving work depending on the care of grandparents and the level of income, 2–3 years of age, average level of education

VARIABLES	(1)	(2)	(3)	(4)	(5)	(6)
	Between 1 and 2 years	Between 1 and 2 years	Between 1 and 2 years	Between 1 and 2 years	Between 1 and 2 years	Between 1 and 2 years
	**reduce working hours**	**reduce working hours**	**reduce working hours**	**leaving work**	**leaving work**	**leaving work**
	**low studies**	**medium studies**	**high studies**	**low studies**	**medium studies**	**high studies**
No income	0.320 ***	0.373 ***	0.410 ***	0.397 ***	0.250 ***	0.151 ***
	(0.102)	(0.089)	(0.096)	(0.094)	(0.061)	(0.049)
<9000 eur	0.308 ***	0.334 ***	0.350 ***	0.290 ***	0.167 ***	0.095 ***
	(0.076)	(0.048)	(0.055)	(0.068)	(0.035)	(0.028)
9000–14,000 eur	0.311 ***	0.338 ***	0.356 ***	0.294 ***	0.170 ***	0.097 ***
	(0.077)	(0.045)	(0.050)	(0.068)	(0.034)	(0.028)
14,000–19,000 eur	0.299 ***	0.305 ***	0.308 ***	0.199 ***	0.106 ***	0.058 ***
	(0.079)	(0.043)	(0.040)	(0.057)	(0.026)	(0.018)
19,000–25,000 eur	0.195 ***	0.210 ***	0.220 ***	0.303 ***	0.174 ***	0.098 ***
	(0.062)	(0.039)	(0.038)	(0.075)	(0.040)	(0.025)
>25,000	0.191 ***	0.190 ***	0.189 ***	0.181 **	0.094 **	0.050 **
	(0.065)	(0.045)	(0.041)	(0.073)	(0.039)	(0.022)
Observations	940	940	940	940	940	940
VARIABLES	(7)	(8)	(9)	(10)	(11)	(12)
	Between 2 and 3 years	Between 2 and 3 years	Between 2 and 3 years	Between 2 and 3 years	Between 2 and 3 years	Between 2 and 3 years
	**reduce working hours**	**reduce working hours**	**reduce working hours**	**leaving work**	**leaving work**	**leaving work**
	**low studies**	**medium studies**	**high studies**	**low studies**	**medium studies**	**high studies**
No income	0.348 ***	0.388 ***	0.325 ***	0.343 ***	0.155 ***	0.172 ***
	(0.114)	(0.104)	(0.098)	(0.105)	(0.054)	(0.065)
<9000 eur	0.322 ***	0.347 ***	0.288 ***	0.317 ***	0.138 ***	0.152 ***
	(0.087)	(0.053)	(0.057)	(0.090)	(0.035)	(0.046)
9000–14,000 eur	0.307 ***	0.317 ***	0.261 ***	0.279 ***	0.116 ***	0.127 ***
	(0.082)	(0.049)	(0.049)	(0.082)	(0.031)	(0.039)
14,000–19,000 eur	0.328 ***	0.312 ***	0.256 ***	0.178 ***	0.067 ***	0.073 ***
	(0.086)	(0.049)	(0.042)	(0.062)	(0.020)	(0.024)
19,000–25,000 eur	0.259 ***	0.254 ***	0.206 ***	0.237 ***	0.092 ***	0.100 ***
	(0.080)	(0.050)	(0.040)	(0.082)	(0.032)	(0.028)
>25,000 (base)	0.155 **	0.146 ***	0.114 ***	0.227 **	0.085 **	0.089 ***
	(0.065)	(0.047)	(0.036)	(0.092)	(0.036)	(0.034)
Observations	744	744	744	744	744	744
VARIABLES	(7)	(8)	(9)	(10)	(11)	(12)
	Between 2 and 3 years	Between 2 and 3 years	Between 2 and 3 years	Between 2 and 3 years	Between 2 and 3 years	Between 2 and 3 years
	**reduce working hours**	**reduce working hours**	**reduce working hours**	**leaving work**	**leaving work**	**leaving work**
	1 child	2 children	3 children or more	1 child	2 children	3 children or more
No income	0.466***	0.355 ***	0.216 **	0.135 **	0.157 ***	0.230 **
	(0.115)	(0.105)	(0.096)	(0.055)	(0.055)	(0.093)
<9000 eur	0.422 ***	0.315 ***	0.188 ***	0.122 ***	0.139 ***	0.201 ***
	(0.063)	(0.054)	(0.070)	(0.036)	(0.036)	(0.073)
9000–14,000 eur	0.389 ***	0.286 ***	0.169 ***	0.103 ***	0.116 ***	0.167 ***
	(0.059)	(0.050)	(0.062)	(0.030)	(0.034)	(0.058)
14,000–19,000 eur	0.383 ***	0.280 ***	0.168 ***	0.059 ***	0.067 ***	0.097 **
	(0.060)	(0.051)	(0.061)	(0.020)	(0.022)	(0.039)
19,000–25,000 eur	0.318 ***	0.226 ***	0.130 **	0.085 ***	0.092 ***	0.129 **
	(0.061)	(0.051)	(0.052)	(0.031)	(0.033)	(0.061)
>25,000 (base)	0.188 **	0.126 ***	0.069 *	0.081 **	0.083 **	0.111 *
	(0.059)	(0.043)	(0.035)	(0.035)	(0.038)	(0.058)
Observations	744	744	744	744	744	744

VARIABLES	(22)	(23)	(20)	(21)
	Between 2 and 3 years	Between 2 and 3 years	Between 2 and 3 years	Between 2 and 3 years
	reduce working hours	reduce working hours	leaving work	leaving work
	medium studies	medium studies	medium studies	medium studies
	no grandparents	yes grandparents	no grandparents	yes grandparents
No income	0.398 ***	0.349 ***	0.173 ***	0.087 **
	(0.105)	(0.110)	(0.060)	(0.039)
<9000 eur	0.358 ***	0.305 ***	0.154 ***	0.075 ***
	(0.055)	(0.061)	(0.040)	(0.026)
9000–14,000 eur	0.329 ***	0.272 ***	0.130 ***	0.061 **
	(0.051)	(0.056)	(0.034)	(0.025)
14,000–19,000 eur	0.326 ***	0.258 ***	0.075 ***	0.034 ***
	(0.051)	(0.057)	(0.023)	(0.013)
19,000–25,000 eur	0.266 ***	0.210 ***	0.104 ***	0.047 **
	(0.052)	(0.054)	(0.036)	(0.020)
>25,000 (base)	0.154 ***	0.115 ***	0.096 **	0.041 **
	(0.049)	(0.043)	(0.041)	(0.021)
Observations	744	744	744	744

Standard errors in parentheses.

***p < 0.01, **p < 0.05, *p < 0.1.

## Footnotes

1 For the unequal effects of the pandemic on employment, see, for example, the report “Women in Work Index 2021” in https://www.pwc.es/es/publicaciones/diversidad/women-in-work-2021-report.pdf and also the reference https://www.brookings.edu/blog/up-front/2021/04/22/class-notes-covid-19-and-womens-labor-the-care-economy-and-more/.

2 This division of labour in the household has been explained mainly in three ways or hypotheses. The first way is based on the specialization of each parent, in accordance with the goal of productive efficiency following the approach of the *new family economy* (Becker, 1981) [13]. That is, the family unit would divide its time between the labour market and domestic work, so that each member would specialize in the activity in which they have the highest productivity. The second way also has to do with the specialization of each parent that would result from an intra-family negotiation process. Domestic work has benefits within the house but also entails an opportunity cost for the person who does it. Each member of the couple can compensate the other for doing this housework, tackling the difference between their work income and that of their partner, which determines their bargaining power. A third way emphasizes the influence of social values and psychological aspects, such as identity, following the approach of *identity economics* (Akerlof and Kranton, 2000) [14]. At the micro level, individuals internalize the gender expectations that others project onto them and act accordingly. At the macro level, the social context, social norms, ethical principles, patriarchal relations, institutions, among others, influence the assignment of work within the home.

3 For example, see: Kleven, H. et al. (2019) [40] and de Quinto, A. et al. (2020) [41].

4 In the sample, the percentage of families using innovative formal care services is around 6% including family spaces, shared parenting groups and day mothers.

5 See: https://ec.europa.eu/social/main.jsp?langId=en&catId=89&newsId=9438&furtherNews=yes.

6 Royal Decree-Law 2/2023, of March 16, on urgent measures for the extension of pensioners' rights, the reduction of the gender gap and the establishment of a new framework for the sustainability of the public pension system (Official State Gazette number 65, March 17/23).
